# Adrenergic receptor signaling induced by Klf15, a regulator of regeneration enhancer, promotes kidney reconstruction

**DOI:** 10.1073/pnas.2204338119

**Published:** 2022-08-08

**Authors:** Nanoka Suzuki, Akinori Kanai, Yutaka Suzuki, Hajime Ogino, Haruki Ochi

**Affiliations:** ^a^Institute for Promotion of Medical Science Research, Faculty of Medicine, Yamagata University, Yamagata 990-9585, Japan;; ^b^Amphibian Research Center / Graduate School of Integrated Sciences for Life, Hiroshima University, Higashi-Hiroshima, Hiroshima 739-8526, Japan;; ^c^Amphibian Research Center, Hiroshima University, Higashi-Hiroshima, Hiroshima 739-8526, Japan;; ^d^Division of Systems Genomics, Department of Computational Biology and Medical Sciences, Graduate School of Frontier Sciences, The University of Tokyo, Kashiwa, Chiba 277-8561, Japan

**Keywords:** regeneration enhancer, kidney, Klf transcription factor, adrenergic receptor, *Xenopus laevis*

## Abstract

Despite the recent discovery of tissue regeneration enhancers in highly regenerative animals, upstream and downstream genetic programs connected by these enhancers still remain unclear. Here, we performed a genome-wide analysis of enhancers and associated genes in regenerating nephric tubules of *Xenopus laevis*. Putative enhancers were identified using assay for transposase-accessible chromatin with high-throughput sequencing (ATAC-seq) and H3K27ac chromatin immunoprecipitation sequencing (ChIP-seq) analyses. Their target genes were predicted based on their proximity to enhancers on genomic DNA and consistency of their transcriptome profiles to ATAC-seq/ChIP-seq profiles of the enhancers. Motif enrichment analysis identified the central role of Krüppel-like factors (Klf) in the enhancer. Klf15, a member of the Klf family, directly binds enhancers and stimulates expression of regenerative genes, including *adrenoreceptor alpha 1A* (*adra1a*), whereas inhibition of Klf15 activity results in failure of nephric tubule regeneration. Moreover, pharmacological inhibition of Adra1a-signaling suppresses nephric tubule regeneration, while its activation promotes nephric tubule regeneration and restores organ size. These results indicate that Klf15-dependent adrenergic receptor signaling through regeneration enhancers plays a central role in the genetic network for kidney regeneration.

## Significance

The kidney is an essential organ for filtrating metabolic waste products, and failure of this function leads to devastating disease. One possible cure is to regenerate functional tissue by reactivating intrinsic genetic programs for kidney formation. Here, we show that Krüppel-like factor 15 (Klf15) coregulates regeneration enhancers identified from the assay for transposase-accessible chromatin with high-throughput sequencing (ATAC-seq) and H3K27ac landscape. We also showed the adrenergic receptor gene is a downstream target of Klf15 and treatment with an agonist for this receptor stimulates nephric tubule regeneration and restores organ size. These results indicate the central role for Klf15-dependent adrenergic receptor signaling in the regeneration program and provide a new pharmacological target for regenerative therapy of kidney disease.

Amphibians and fish have been valuable model systems for studying kidney development, diseases, and regeneration (1, 2). The nephron structure and genetic pathway that regulates nephrogenesis are conserved among vertebrates, providing insight into human regeneration. In humans and mice, surviving tubular epithelial cells are the primary cellular source of the repair process and it is difficult to directly observe the regenerating process under the microscope (3). In *Xenopus laevis*, epithelial cells in nephric tubules are also the primary cellular source of the repair process (4). Unlike mammals, nephrons of *Xenopus* embryos are located just beneath the skin. This allows direct observations of regenerating tubules after their injury, enabling model systems to understand the mechanisms of nephric tubule regeneration.

During regeneration, cells first receive signals from the injured area, then restart proliferation, patterning, and differentiation (5). Hence, embryonic development and tissue/organ regeneration share many characteristic events at the tissue and cellular levels, with some exceptions, such as wound healing, dedifferentiation, and transdifferentiation (6). Recent studies have revealed that numerous developmental genes evolutionarily conserved among vertebrates are reactivated during regeneration (6). These findings imply regenerative capacity is generally governed by gene expression mechanisms rather than presence or absence of genes. Therefore, the *cis-*regulatory mechanisms that regulate the gene expression after injury are key to understanding the molecular basis of regeneration. Among the *cis*-regulatory elements, enhancers are essential in regulating spatiotemporal gene expression (7). Recently, enhancers involved in injury and/or regeneration have been identified using regenerative systems, including the kidney (4, 8, 9). Additionally, transcription factors that directly bind to the injury and/or regeneration enhancers and activate targets gene expression have also been identified (4, 10). Activator protein 1 (AP-1) complex is well known to regulate gene expression in response to various stimuli, such as cytokines, growth factors, and stress signals, and this stress-responsive complex regulates gene expression via the damage-responsive enhancer for *Drosophila* imaginal disks regeneration or the regeneration-responsive enhancers for teleost fin regeneration (10, 11). Arid3a, an AT-rich interaction domain family transcription factor, with H3K9me3 demethylases KDM4/JMJD2 complex modulates H3K9me3 levels on evolutionarily conserved regeneration signal-response enhancers (4). Krüppel-like factor 1 (Klf1) regulates activity of enhancers for zebrafish cardiac regeneration (12). Therefore, emerging evidence suggests identifying enhancers involved in injury and/or regeneration and their input transcription factors is a straightforward approach to reveal the fundamental molecular mechanisms behind tissue regeneration. Nonetheless, the number of enhancers and their input transcription factors identified to date are still limited and the gene regulatory networks wired through *cis*-regulatory elements for regeneration have yet to be fully investigated.

Assay for transposase-accessible chromatin with high-throughput sequencing (ATAC-seq) is widely used to identify open chromatin regions (13). The acetylation of histone H3 at lysine 27 (H3K27ac) is a well-defined marker of active enhancer, and is required for enhancer function (14, 15). Recent progress shows that profiling of ATAC-seq and H3K27ac chromatin immunoprecipitation sequencing (ChIP-seq) data identified many putative enhancers involved in injury and/or regeneration (11, 12, 16). Here, we profiled genome-wide changes in chromatin accessibility (ATAC-seq), H3K27ac modification, and gene expression (RNA-seq) during *X. laevis* nephric tubule regeneration. These profiles and further molecular analyses showed that Klf15 functions as an input transcription factor for injury- and/or regeneration-associated enhancers and *alpha-1A adrenergic receptor* (adra1a/α1-adrenoreceptor [AR]) is one of the Klf15 target genes. Further, engrailed repressor domain (EnR) fused-Klf15 and pharmacological blocking of Adra1a using prazosin suppresses nephric tubule regeneration. In contrast, treatment with agonists for adrenergic receptors promotes extension of regenerating nephric tubules and restores nephron size. Therefore, this study offers a link between injury-responding transcription factor and target genes via the kidney regeneration enhancer.

## Results

### Kidney Injury Modifies Open Chromatin.

**Fig. 1.**
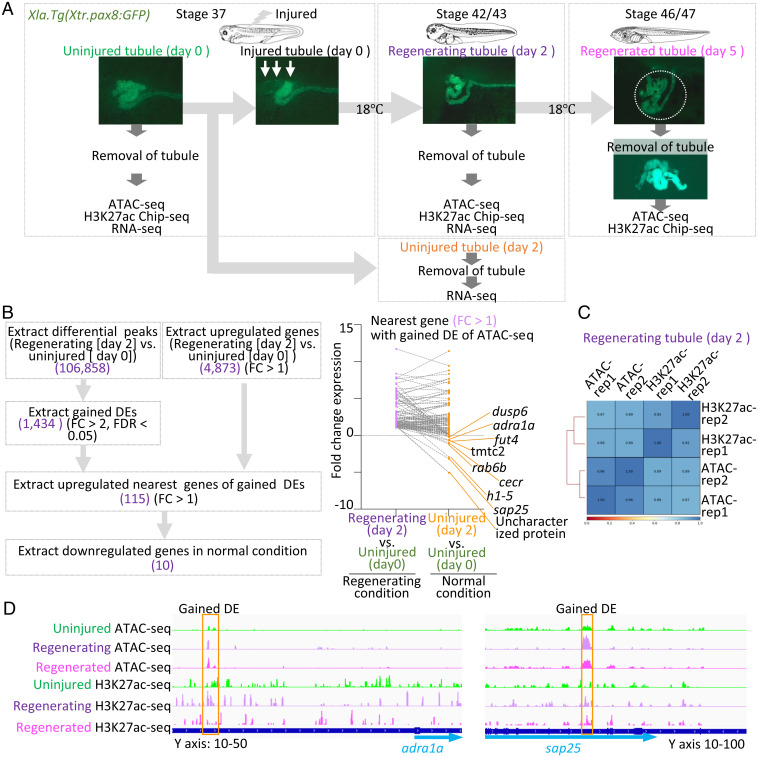
Chromatin accessibility dynamics during *X. laevis* nephric tubule regeneration. (*A*) Schematic illustration of experimental design for ATAC-seq, H3K27ac ChIP-seq, and RNA-seq. Pax8:GFP positive proximal and intermediate tubules were collected using tweezers from uninjured (day 0), regenerating (day 2), and regenerated (day 5) conditions for ATAC-seq and H3K27ac ChIP-seq. Proximal and intermediate tubules were collected from uninjured (day 0), uninjured (day 2), and regenerating (day 2) conditions for RNA-seq. (*B*) Upregulated genes nearest to the gained DEs in regenerating nephric tubule. Genes nearest to the gained DEs were extracted using ChIPpeakAnno. Up-regulated genes were then extracted (purple circles above the dotted line). Their counterpart genes in normal conditions (uninjured tubules [day 2] vs. uninjured tubules [day 0]) are plotted as orange circles. (*C*) Correlation analysis of ATAC-seq and ChIP-seq peaks. Hierarchically clustered correlation matrix of ATAC-seq and ChIP-seq peaks and their replicates (rep1 and rep2). (*D*) Gained DEs and H3K27ac-seq tracks.

To identify the enhancers involved in kidney regeneration, we used a transgenic line of *X. laevis Xla.Tg(Xtr.pax8:GFP)*, which visualizes regenerating nephric tubules (4, 17). We first performed ATAC-seq, ChIP-seq for H3K27ac (marker for active enhancers), and RNA sequencing (RNA-seq) (13, 18). After injury of proximal tubules, cells in the remaining tubules begin to express developmental genes involved in kidney regeneration, such as *lhx1* and *pax8,* within 2 d, then regenerated functional nephric tubules within 5 d after injury (Fig. 1*A*) (4). During regeneration, the proximal and intermediate tubules cells proliferate and migrate into regions where cells are removed (4). Therefore, to chart the enhancer landscape for *Xenopus* nephric tubule regeneration, we chose to use proximal and intermediate tubules extracted from uninjured (day 0), regenerating (day 2), and regenerated conditions (day 5). Since surgical removal of nephric tubules in *Xenopus* is simple and easy, we collected nephric tubules using tweezers under the fluorescence microscope (Fig. 1*A*).

To obtain putative active enhancers in regenerating nephric tubules, we first searched genomic elements displaying increased accessibility during regeneration. We used the edgeR software package to identify the differential open chromatin peaks between regenerating tubules (day 2) and uninjured tubules (day 0) (19). Among 106,858 differentia peaks, 1,434 elements were significantly increased accessibility, gained differential elements (DEs), in the regenerating tubules with false discovery rate <5% and fold change >2. To predict target genes of gained DEs in regenerating nephric tubules, we analyzed transcriptome data and found 4,873 genes were up-regulated in regenerating nephric tubules (regenerating tubule [day 2] vs. uninjured tubule [day 0]). Lhx1 and Pax8 are well known to regulate kidney development, and their expression is induced immediately after a nephrectomy (4, 20, 21). It was confirmed that *lhx1* and *pax8* were included in up-regulated genes in RNA-seq (Dataset S1). We then extracted up-regulated nearest genes of gained DEs in the regenerating condition utilizing ChIPpeakAnno (22). Among 4,873 up-regulated genes, 115 are located near the gained DEs (Fig. 1*B*, purple circles). To confirm whether these up-regulations depend on injury, we compared counterpart genes in normal conditions (uninjured tubules [day2] vs. uninjured tubules [day 0]; Fig. 1*B*, orange circles). Genes suppressed in uninjured tubules (day 2) compared with uninjured tubules (day 0) may be unnecessary in normal conditions of stage 42/43 nephric tubules (Fig. 1*B*, orange circles, fold-change < 0). Among the 115 up-regulated nearest genes of gained DEs, we focused on genes that satisfy the following criteria: genes suppressed in normal conditions and up-regulated in regenerating conditions (Fig. 1*B* and *SI Appendix*, Fig. S1*B*). These genes and their gained DEs may provide us with the link between injury-responding transcription factor and target genes via kidney regeneration enhancers. The above criteria allowed us to find *adra1a* (*adrenoreceptor alpha 1a*), *rab6b* (*rab6b, member RAS oncogene family*), *fut4* (*fucosyltransferase 4*), *sap25* (*sin3a associated protein 25*), uncharacterized protein (LOC108716608), *h1-5* (*h1.5 linker histone, cluster member*), tmtc2 (tr*ansmembrane O-mannosyltransferase targeting cadherins 2*), and *dusp6* (*dual specificity phosphatase 6 S homeolog*) (*SI Appendix*, Figs. S1*B* and S2). Previous studies showed that ATAC-seq and H3K27ac overlapped peaks often associated with regeneration enhancers (16). As expected, peaks of ATAC-seq were highly correlated with H3K27ac ChIP-seq peaks (Fig. 1 *C* and *D* and *SI Appendix*, Figs. S1*A* and S3). Therefore, we obtained elements displaying increased accessibility during regeneration, putative regeneration enhancers, and their candidate target genes induced by the injury.

### Klf15 Functions as a Transcription Activator for Open Chromatin Elements.

**Fig. 2.**
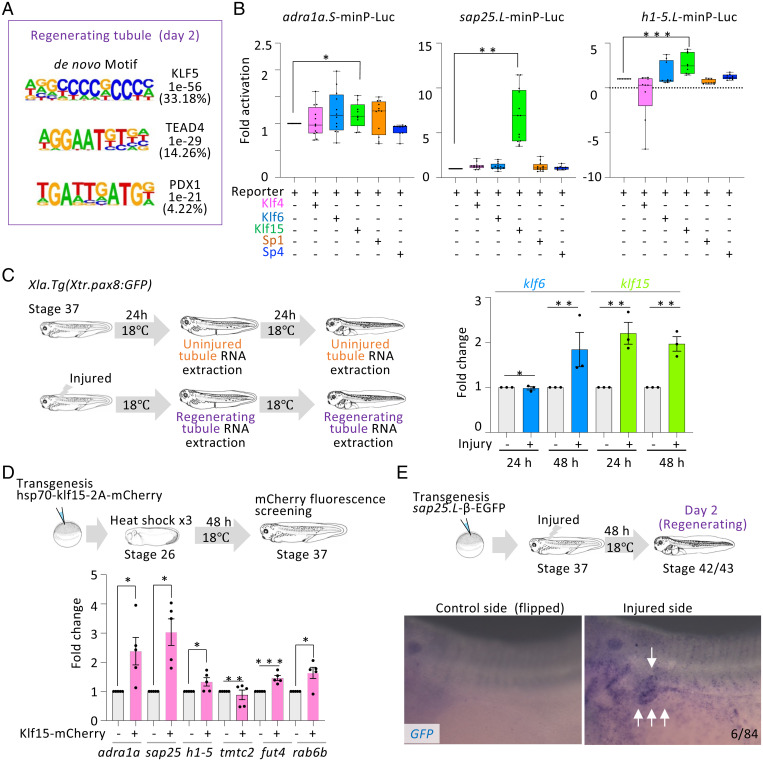
Klf15 is a transcriptional activator for regeneration enhancers. (*A*) *de novo* identification of regenerating specific open chromatin elements and best matches for known motifs. *P* values for motif enrichment and percentage of regions containing motifs are indicated to the right of the sequence logos. (*B*) The luciferase reporter assay for gained DE near *adra1a.S*, *sap25.L*, and *h1-5.L* was performed using HEK293T cells. Klf4, Klf6, Klf15, SP1, and SP4 were cotransfected with reporter plasmids. Two-tailed unpaired Mann-Whitney *t* test: *P = 0.0154; **P = 0.0001; ***P < 0.0001. Whiskers show minimum and maximum and all points. (*C*) Expression of *klf15* was induced immediately after injury. qPCR analysis of *klf6* and *klf15* after injury. Expression of *klf6* and *klf15* in uninjured nephric tubules set to 1, respectively. Significant differences between uninjured and injured were calculated using two-tailed unpaired Mann-Whitney *t* test: *P ≥ 0.9999 (not significant), **P = 0.1. (*D*) Klf15 induces gene expression associated with gained DE. Expression of target genes in heat shocked treated mCherry negative tadpoles set to 1. Significant differences between mCherry negative and positive tadpoles were calculated using two-tailed unpaired Mann-Whitney *t* test: *P = 0.0079, **P = 0.127, ***P = 0.6285. (*E*) Gained DEs in regenerating nephric tubule function as a regeneration enhancer responding to injury signal. GFP reporter construct carrying gained DE near *sap25.L* with the β-actin proximal promoter subjected to transgenesis. All reporter-injected embryos underwent injury on the left side at stage 37. Arrow indicates regenerating nephric tubule and GFP signals. N indicates the number of scored tadpoles, whereby six tadpoles showed a stronger GFP signal on the injured side compared with the uninjured control side.

To identify drivers of the regeneration enhancers, we applied the Hypergeometric Optimization of Motif EnRichment (HOMER) tool, a de novo motif-discovery algorithm well suited to search DNA binding motifs in large-scale chromatin datasets (23). We first extracted regeneration-specific open chromatin elements (*SI Appendix*, Fig. S4*A*), performed HOMER analysis. We found a robust enrichment of Krüppel-like family of transcription factors (KLFs) motifs in regenerating specific elements (Fig. 2*A* and *SI Appendix*, Fig. S4 *B* and *C*). KLF family members are involved in various aspects of gene regulation. KLF4 is known to function as a pioneer factor by binding to the closed chromatin state and converting to open chromatin in reprogramming fibroblast to pluripotency (24). KLFs function as transcriptional activators or repressors, while some KLFs are bifunctional (25). For example, Klf4 possesses both activation and repression domains and functions in a context-dependent manner (25). To investigate which KLFs function as transcriptional activators for nephric tubule regeneration, we searched the RNA-seq data and found that *klf4*, *klf6*, *klf15*, *sp1*, and *sp4* were expressed in regenerating nephric tubules. We cloned the full-length cDNAs of these *klfs* from *Xenopus tropicalis* in expression vectors, and also constructed the luciferase reporters containing the open chromatin elements listed in *SI Appendix*, Fig. S1*B*. Transient cotransfection assays of the KLF-expression vectors and the luciferase reporters in cultured cells showed that Klf6 and Klf15 function as activators for open chromatin elements associated with *adra1a*, *sap25*, and *h1-5*, while Klf4 functions as a repressor for the *h1-5* element (Fig. 2*B*). In contrast, the element near uncharacterized protein gene-LOC108716608 was activated by Klf4 (*SI Appendix*, Fig. S5). The reporters carrying *rab6b*, *fut4*, *A. superbus venom factor 1*, and *tmtc2* elements showed no significant up-regulation by Klf4, Klf6, Klf15, SP1, and SP4 (*SI Appendix*, Fig. S5). Since other KLF families, such as SP8, are also expressed in regenerating nephric tubules, these KLFs may function as activators (Dataset S1). We failed to clone the open chromatin elements associated with *cecr* and *dusp6*. To examine whether expression of *klf6* and *klf15* are induced by injury, we performed qPCR. We found that *klf15* expression was induced within 24 h after the injury, while *klf6* expression appeared at 48 h (Fig. 2*C*). *In situ* hybridization analysis confirmed that *klf15* was expressed in the regenerating nephric tubules after the injury, as in the uninjured nephron as previously reported (26) (*SI Appendix*, Fig. S6*A*). Therefore, Klf15 is one of the candidate transcription factors for activating regeneration-specific open chromatin elements. We then examined whether Klf15 induces expression of the putative target genes, *adra1a*, *sap25*, and *h1-5* in *X. laevis*. Heat shock-inducible Klf15 transgenic founder assay showed that endogenous *adra1a.S*, *sap25.L*, and *h1-5.L* were significantly induced by Klf15 (Fig. 2*D*). We also generated transgenic founder tadpoles carrying a Green fluorescent protein (GFP) reporter gene linked to either the open chromatin element associated with *sap25*, *adra1a*, or *h1-5*. The nephron of the *Xenopus* embryos appeared on both sides. Therefore, we injured the left side of the nephron and used the other side as a control. The resulting tadpoles showed GFP expression in the regenerating nephric tubules on the injured side, suggesting their enhancer activity for the regeneration (Fig. 2*E* and *SI Appendix*, Fig. S6*B*).

### Klf15 Is Critical for Nephric Tubule Regeneration.

**Fig. 3.**
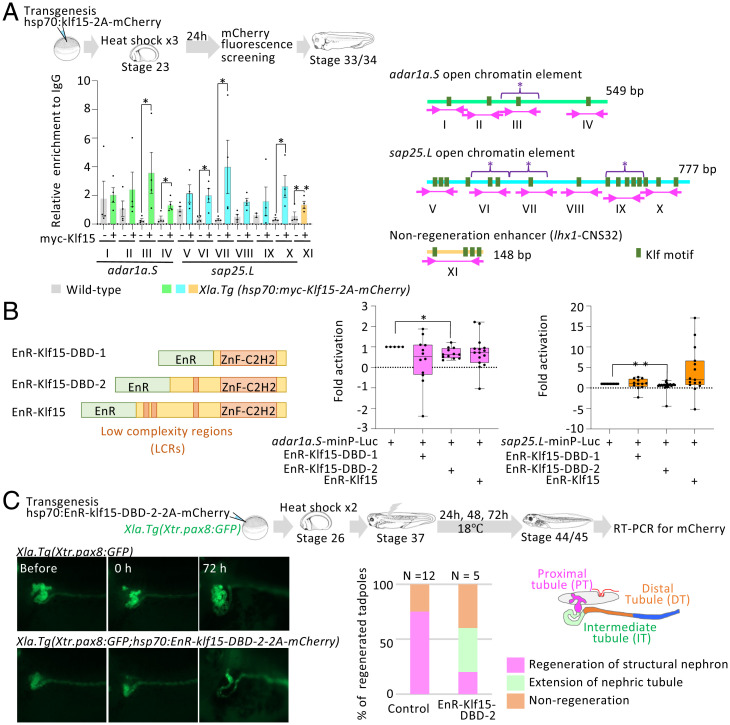
Klf15 is required for nephric tubule regeneration. (*A*) Klf15 directly binds to open chromatin elements. Significant differences were calculated using two-tailed unpaired Mann-Whitney *t* tests: *P < 0.029, **P = 0.3143 (not significant). Error bars indicate mean with SEM. (*Right*) Schematic illustration of amplified region in the open chromatin element and Klf motifs. Asterisks indicate Klf5 binding site. (*B*) EnR domain fused Klf15 functions as a transcriptional repressor for open chromatin elements. Luciferase reporter assay for open chromatin elements near Adra1a and Sap25 (*P = 0.0057, **P ≤ 0.0001). (*C*) Heat shock-treated *Xla.Tg(Xtr.pax8:GFP;hsp70:EnR-klf15-DBD-2-2A-mCherry)* failed to regenerate nephric tubules.

To test Klf15 occupancy on open chromatin elements, we performed ChIP-qPCR using heat shock-inducible Klf15 transgenic founder embryos of *X. laevis*. The JASPAR (version 9) database was used to identify the putative Klf binding site on these elements (Fig. 3*A*) (27). The ChIP-qPCR showed the Klf15 directly binds to the open chromatin element in heat shock-treated *X. laevis*, whereas no enrichment of Klf5 was detected on *lhx1*-CNS32 that showed no enhancer activity in regenerating nephric tubules in our previous study (Fig. 3*A* and *SI Appendix*, Fig. S7*A*) (4). To investigate the potential role of Klf15 in regeneration of nephric tubules, we generated dominant negative constructs of *klf15* that express the engrailed repression domain (EnR) fused to a full-length Klf15 (EnR-Klf15) and the EnR fused to a partial Klf15 lacking its N-terminal low complexity region (EnR-Klf15-DBD-1, EnR-Klf15-DBD-2; Fig. 3*B*). We then tested whether EnR fused Klf15 can suppress expression of the luciferase reporter carrying the open chromatin element of *adra1a* or *sap25.* Among them, EnR-Klf15-DBD-2 efficiently suppressed reporter activities (Fig. 3*B*). Next, we generated heat shock-inducible EnR-Klf15-DBD-2 transgenic founders to examine the effects this construct *in vivo*. When heat-shock was provided, the resulting transgenics failed the nephric tubule regeneration by 72 h after the injury (Fig. 3*C* and *SI Appendix*, Fig. S7*B* and *SI Appendix*, *SI Methods*). This result is reminiscent of our previous finding that the knockdown of a regenerative gene, *arid3a*, results in failure of the nephric tubule regeneration around 72 h after the injury (4). These lines of evidence indicated that Klf15 regulates the nephric tubule regeneration by activating regeneration-specific open chromatin elements as enhancers.

### *adra1a* Targeted by Klf15 Promotes Kidney Regeneration.

**Fig. 4.**
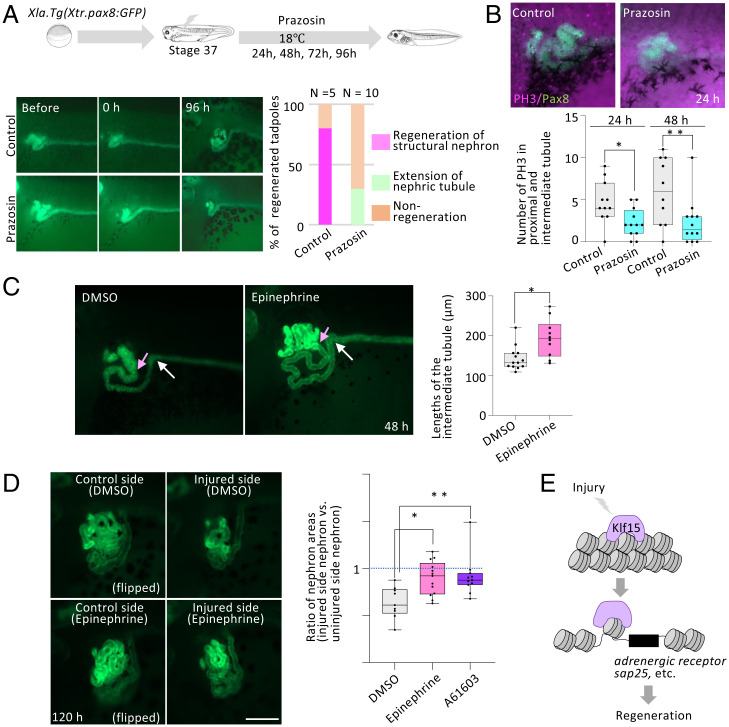
Adra1a promotes kidney regeneration. (*A*) Antagonist for Adra1a suppresses regeneration of nephric tubules. Nephric tubule injured *Xla.Tg(Xtr.pax8:GFP)* tadpoles were treated with prazosin, an agonist for alpha-1 adrenergic receptors. (*B*) The antagonist for Adra1a suppresses cell proliferation in regenerating nephric tubules. Immunofluorescence staining with anti-phosphorylated histone H3 antibody, a marker for mitotic cells. Two-tailed unpaired Mann-Whitney *t* test: *P = 0.0148, **P = 0.0276. (*C*) Epinephrine promoter for elongation of intermediate nephric tubules. Pink arrow to white arrow indicates the intermediate tubule. Significant differences between DMSO and epinephrine treatment was calculated using two-tailed unpaired Mann-Whitney *t* test: *P = 0.0076. (*D*) Nephron size was recovered following treatment with an agonist for adrenergic receptors. Nephric tubule area for injured and uninjured sides was measured to obtain the ratio of regenerated organ size. Two-tailed unpaired Mann-Whitney *t* test: *P = 0.0043, **P = 0.0023. (Scale bar, 50 μm). (*E*) Model illustrating the Klf15 function in kidney reconstruction.

The *adra1a* and *sap25* genes, which were identified as the direct targets of Klf15, may have active roles in the nephric tubule regeneration. Since agonists and antagonists for Adra1a are well characterized, we, therefore, decided to test whether adrenergic receptor contributes to regeneration of nephric tubules (28). Prazosin is a selective blocker for alpha-1 adrenergic receptor and has been previously applied to zebrafish (28, 29). We injured the nephric tubules of transgenic *Xenopus* tadpoles (*Xla.Tg(Xtr.pax8:GFP)*) at stage 37 and treated them with 0.04 mg/mL prazosin, which resulted in inhibition of the nephric tubule regeneration (Fig. 4*A*). During proximal tubule regeneration, apoptosis occurs within 3 h after injury and the number of apoptotic cells decreased within 1 d (30). Meanwhile, nephric tubules extend from remaining tubules accompanied by cell proliferation (4). Therefore, we investigated whether cell proliferation was affected by treatment of prazosin. Immunofluorescence staining with anti-phosphorylated histone H3 antibody (PH3), a marker for mitotic cells, showed that the number of PH3 positive cells in GFP positive nephric tubules significantly decreased at 24 h and 48 h in the prazosin-treated tadpoles (Fig. 4*B*). We then explored whether agonists for adrenergic receptors promote nephric tubule regeneration. Epinephrine activates alpha-1, alpha-2, beta-1, and beta-2 adrenergic receptors and A-61603 is a selective α1A-adrenergic receptor. As in the case of prazosin, we injured the nephric tubules of the transgenic *Xenopus* tadpoles, treated them with these agonists, and found that proximal tubules and intermediate tubules were elongated compared with those treated with dimethyl sulfoxide (DMSO) alone (control) (Fig. 4*C*). To quantify the effect of the agonist, we measured the length of the intermediate tubule (Fig. 4*C*). Although proximal tubules were also elongated, it was difficult to measure the length, since it already formed the complex structure (Fig. 4*C*). We generally injured the left side and used the other side as a control. Although the injured nephric tubule regenerates, the size of the regenerated tubule is slightly smaller than the uninjured control side (Fig. 4*D*). Since Adra1a activation promotes nephric tubule regeneration, we examined whether the size of the regenerated tubules was restored. We measured nephric tubule area and found that the size of the regenerated tubule was restored following treatment with both epinephrine and A-61603 agonist (Fig. 4*D*). On the other hand, we examined whether adrenergic signal modulation affects normal nephrogenesis in the absence of injury and found that adrenergic signaling primarily functions in regenerating nephric tubules (*SI Appendix*, Fig. S8). Thus, the target of Klf15, the adrenergic receptor, is crucial for successful kidney regeneration.

## Discussion

Many developmental genes are reactivated for regeneration and such genes are evolutionarily conserved among vertebrates. Therefore, understanding *cis*-regulatory mechanisms of highly regenerative animals offers insight into the regenerative capacity of animals. Our studies on identifying regeneration enhancers uncover an unexpected role of Klf15 and its target adrenergic receptor during nephric tubule regeneration (Fig. 4*E*).

KLF and SP belong to the family of transcription factors that possess zinc finger DNA binding domain and are involved in various processes. KLF4, a Yamanaka factor, is a crucial constituent of reprogramming (31). KLF4, OCT4, and SOX2 can bind to nucleosomes, and a recent study showed that KLF4 forms a liquid-liquid phase separation with DNA that recruits OCT4 and SOX2 (24, 32). We showed that Klf15 and Klf6 function as transcriptional activators for the *adra1a* and *sap25* regeneration enhancer, while Klf4 also functions as transcriptional activators for the uncharacterized protein gene (LOC108716608) enhancer. Therefore, KLFs may act as activators for regeneration enhancers in a context-dependent manner. To date, no evidence identifies that Klf15 binds to the nucleosome in nephric tubule cells and it is possible that Klf15 functions as a pioneer factor for kidney regeneration. Further studies on chromatin opening mechanisms by Klf15 may provide novel insights into the initial step of genomic dynamics that occur immediately after the kidney injury for the subsequent regeneration.

Adra1a, one of the targets for Klf15, is a α1-AR. ARs are G protein coupled receptors that bind endogenous epinephrine, norepinephrine, and catecholamines. The biological function of adrenergic receptors is well known in regulating the sympathetic nervous system (28). In addition, previous studies show that stress-induced local epinephrine delays wound healing through the β2-AR (33, 34). Here, we showed that treatment of adrenergic receptor agonists promotes extension of proximal and intermediate tubules during regeneration and that this extension restores organ size. In general, wound healing and blastema formation following injury are the first steps for successful regeneration and the blastema acts as a signaling hub for subsequent cell proliferation and dedifferentiation (6). We have not examined whether epinephrine delays wound healing of injured nephric tubules. It is possible that ARs are involved in various regeneration processes. Further step-by-step analyses are required to reveal the detailed function of ARs in regenerating kidneys.

Sin3A associated protein 25 (SAP25) has been identified as a binding protein of the transcriptional corepressor mSin3, which is associated with histone deacetylase. SAP25 accumulates in promyelocytic leukemia protein (PML) nuclear bodies depending on H-RAS induced senescence (35). The PML body mediates various stress signaling pathways, such as cytokine, hypoxia, heat shock, and DNA damage (36). Sin3A is located near gained DE and slightly up-regulates under regenerating conditions (Dataset S2). In addition, a previous study showed that the repressor type of SP transcription factor directly interacts with Sin3A (37). Although the functional roles of SAP25 in regeneration are still unclear, SAP25 may accumulate in the PML body with Sin3a and SPs in response to injury signals or subsequent regeneration signals to contribute to the kidney regeneration. Further studies on SAP25 may reveal the roles for accumulating the transcriptional repressor complex in the PML body during regeneration.

Here, we identified the genes closely associated with the gained DEs using the available algorithm ChIPpeakAnno. However, since enhancers can regulate the gene expression within the topologically associating domain (TAD), a larger number of genes may be under the regulation of the gained DEs in regenerating nephric tubules (38). Future analysis of TADs in regenerating nephric tubules is expected to identify such new regenerative genes.

## Materials and Methods

### Injury of *Xenopus* Nephric Tubule.

We previously reported the surgical removal of the nephric tubule using stage 37 *Xla.Tg(Xtr.pax8:GFP)* embryos in accordance with McLaughlin’s method (4, 30). All injured *X. laevis* were cultured at 18 °C.

### ATAC-seq and ChIP-seq Library Construction and Mapping.

In brief, 50 nephric proximal and intermediate tubules per sample were collected and cells were dissociated by incubation with liberase TM (Roche) for 5 min at 37 °C. Cells for ATAC-seq were then subjected to the OmniATAC-seq protocol, as previously described (38). Library construction and sequencing on an NovaSeq (Paired-end 125 bp) were performed by the Platform for Advanced Genome Science. ChIP-seq libraries were prepared using the ThruPLEX DNA-Seq Kit (Takara Bio In.) and sequenced on an NovaSeq (Paired-end 150bp). Reads were mapped onto the *X. laevis* genome sequence assembly (GCF_001663975.1_Xenopus_laevis_v2). Detailed methods are provided in the *SI Appendix*, *Methods*.

### Differential Peak Analysis of ATAC-Seq and ChIP-Seq.

For differential ATAC-seq peaks, narrow peaks were obtained using MACS2 (2.2.6). Peaks in independent samples were merged and fragments per peak in each sample were counted using featureCounts (2.0.1) and edgeR (3.32.1) software packages to detect differential ATAC-seq peaks (RRID: SCR_012919) (RRID:SCR_012802) (19, 39). R studio (4.0.4) was used to run R scripts. For differential H3K27ac ChIP-seq peaks, we applied broad peak calling on H3K27ac marks. MACS2 module bdgdiff with default parameters was used to identify differential peaks. IGV genome browser was utilized to visualize peaks (2.12.2) (40).

### Motif Enrichment Analysis.

Unique and overlapping peaks were computed using BEDTools (2.30.0) (RRID:SCR_006646) (41). *De novo* identification of transcription factor motifs enriched in open chromatin element was performed with HOMER tools (RRID:SCR_010881; parameters: -size 200 -mask) (23).

### Transgenesis of *X. laevis* and Reporter Assay.

Transgenesis procedures of *X. laevis* were previously described (42). In brief, GFP reporter constructs carrying open chromatin elements with β-actin proximal promoter were subjected to transgenesis (43). All reporter-injected embryos underwent injury on the left side at stage 37. We performed in situ hybridization to examine GFP expression with maximum sensitivity using all normally-developed tadpoles. Detailed methods are provided in the *SI Appendix*, *SI Methods*.

### Heat Shock of Transgenic *Xenopus.*

*Xla.Tg(Xtr.pax8:GFP;hsp70:klf15-2A-mCherry)* transgenic *X. laevis* at tailbud stage 26 were treated at 34 °C for 15 min, followed by 15 min at 14 °C. These steps were repeated three times and embryos were incubated at 18 °C. Normally developed heat shock-treated embryos were then sorted by mCherry positivity or negativity. *Xla.Tg(Xtr.pax8:GFP;hsp70:EnR-klf15-DBD2-2A-mCherry)* transgenic *X. laevis* were treated two cycles of 34 °C for 15 min and 14 °C for 15 min. Embryos were subjected to RT-PCR to confirm expression of *mCherry* (*SI Appendix*, Fig. S6*B*). Detailed methods are provided in the *SI Appendix*, *SI Methods*.

### Agonist and Antagonist Treatment for Adra1a.

Injured embryos were incubated in 0.04 mg/mL prazosin hydrochloride (Sigma-Aldrich), 100 μg/mL (-) -Epinephrine (Sigma-Aldrich), and 100 nM A-61603 (Cayman) at 18 °C. Buffer was exchanged every 24 h. Detailed methods are provided in the *SI Appendix*, *Methods*.

## Supplementary Material

Supplementary File

Supplementary File

Supplementary File

## Data Availability

ATAC-seq, ChIP-seq, and RNA-seq data have been deposited in the DDBJ BioProject database (https://www.ddbj.nig.ac.jp/dra/index-e.html) (accession numbers PRJDB9147 (44) and PRJDB13124 (45)).
