# Supplementary material for: Adrenergic receptor signaling induced by Klf15, a regulator of regeneration enhancer, promotes kidney reconstruction
Source: Proc Natl Acad Sci U S A. 2022 Aug 8;119(33):e2204338119. doi: 10.1073/pnas.2204338119 (PMC9388080; doi:10.1073/pnas.2204338119)
Supplement: Supplementary File [file pnas.2204338119.sapp.pdf]

Supplementary Information for
Adrenergic receptor signaling induced by Klf15, a regulator of regeneration enhancer, promotes kidney reconstruction

Nanoka Suzuki, Hajime Ogino and Haruki Ochi*

Haruki Ochi
Email: harukiochi@med.id.yamagata-u.ac.jp

This PDF file includes:

Supplementary text: Supplementary Materials and Methods.
Figures S1 to S8
SI References

Other supplementary materials for this manuscript include the following:

Datasets S1 to S3

Supplementary Information Text

SI Methods

ATAC-seq library construction and mapping

Two replicates were used for all ATAC-seq experiments. Proximal and intermediate tubules of *Xla.Tg(Xtr.pax8:GFP)* were extracted from uninjured (day 0), regenerating (day2), and regenerated (day 5) conditions under fluorescence microscope. Fifty nephric tubules per sample were collected and cells were dissociated by incubation with 1 mg/ml liberase TM (Roche, Cat#5401127001) for 5 min at 37°C. Cells were collected by centrifuge for 5 min at 4,000 g, and then stored in CELLBANKER 1 (Takara Bio In., Cat#CB013). Cells were then subjected to the OmniATAC-seq protocol, as previously described (1). Construction of libraries and sequencing was performed by the Platform for Advanced Genome Science (PAGS) using NovaSeq, 125bp Paired-end seq. We achieved an average of 228 million total reads per ATAC-seq library in this study, varying between 36 and 118 million reads for each library. Reads were mapped onto the *Xenopus laevis* genome sequence assembly (GCF_001663975.1_Xenopus_laevis_v2), obtained from the NCBI site using boweite2 (2.41) with default parameters. ATAC-seq peaks were called with MACS2 (2.2.6) (parameters: --nomodel --g 3e9 --f BAM -nonmodel --B --p 0.01).

ChIP-seq library construction and mapping

50 nephric proximal and intermediate tubules per sample were collected and cells were dissociated by incubation with liberase TM for 5 min at 37°C. Cells were stored in CELLBANKER 1, then treated with 200U/μL Micrococcal Nuclease (MNase) for 450 sec at 37℃ (NEB, Cat#M0247) (2). We then added 10% of the volume of 100 uM EDTA followed by mixing, then added 10% of the volume of 1% Triton/1% deoxycholate solution. A 1:500 Rabbit polyclonal H3K27ac antibody (Abcam, Cat#ab4729, RRID: AB_2118291) was added to the MNase digested chromatin and incubated overnight at 4ºC on a rotating platform. We retained 1% of chromatin used for each ChIP reaction as input DNA. Protein A Dynabeads were added to ChIP reactions and incubated for an additional 4 hours at 4°C. Magnetic beads were washed, followed by reversal of crosslinks and DNA purification. ChIP-seq libraries were prepared using the ThruPLEX DNA-Seq Kit (Takara Bio In., Cat# R400674) and sequenced on an Illumina NovaSeq (PE150). Reads were mapped onto the *Xenopus laevis* genome sequence assembly using boweite2 (2.41) with default parameters. Chip-peaks were called with MACS2 (2.2.6) (parameters: --nomodel --g 3e9 --f BAM -nonmodel --B --p 0.01).

RNA-seq library generation and mapping

50 proximal and intermediate tubules per sample were collected and total RNA was purified using PureLink RNA min kit (Thermo Fisher Scientific, Cat#12183018A). RiboMinus Eukaryote Kit for RNA-Seq (Thermo Fisher Scientific, Cat#A1083708) was used to deplete ribosomal RNA. Libraries were prepared using the Ion AmpliSeq Library Kit Plus (Thermo Fisher Scientific, Cat#4488990) and sequenced on an Ion Torrent. Reads were mapped onto the *Xenopus laevis* genome sequence assembly using HISAT2 (2.2.1) with default parameters (RRID: SCR_015530). Differential expression analysis was performed with default parameters using R package DEseq2 (1.30.1).

Correlation between ATAC-seq and ChIP-seq peaks

To compare ATAC-seq and ChIP-seq peaks, read coverage across regions of interest (ATAC-seq and H3K27ac) was analyzed using deepTools (3.5.0) module multiBamSummary and plotCorrelation function with default parameters (RRID:SCR_016366) (3). For each condition, biological replicates correlated well with each other (Pearson correlation coefficient > 0.85).

DNA constructs

PCR products of *Xenopus tropicalis klf6*, *klf4*, *klf15*, *sp1*, and *sp4* were introduced into Xho I and Xba I sites of the pCS2 + MT plasmid. For luciferase reporter assay, PCR products of *Xenopus laevis* open chromatin elements were introduced into Kpn I and Hind III sites of the pGL4.23 vector (Promega, Cat#E8411). For *X. laevis* transgenic reporter analysis, PCR products of *Xenopus laevis* (J strain) open chromatin elements were introduced into the Not I and Bam HI sites of the ISβEGFP vector, which contained chicken β-actin basal promoter (4). Engrailed

repressor domain (EnR) fused Klf15 vectors were constructed using PCR amplified EnR and Klf15 full-length or DNA binding domains. DNA fragments were introduced into XhoI and XbaI sites of the pCS-MT vector. To generate heat shock inducible transgenic *X. laevis*, PCR amplified *myc-klf15* and *2A-mCherry* were introduced into Eco RI and Xba I sites of the IS-hsp70 cloning vector (2, 12). In the same context, EnR-Klf15 and 2A-mCherry was introduced into Eco RI and Xba I sites of the IS-hsp70 cloning vector. DNA fragments were constructed using the InFusion cloning method (Takara Bio In., Cat# 639648). Primers used for vector construction are listed in Supplemental Dataset 3.

Transgenesis of *X. laevis* and reporter assay

Transgenic *X. laevis* were generated via the sperm nuclear transplantation method with oocyte extracts (7). GFP reporter constructs carrying open chromatin elements with β-actin proximal promoter were subjected to transgenesis. All reporter-injected embryos underwent injury on the left side at stage 37. We performed *in situ* hybridization to examine GFP expression with maximum sensitivity using all normally-developed tadpoles. All open chromatin element carrying reporters were tested at least three times. The image shows a representative expression pattern of GFP in regenerating nephric tubules. The frequency of GFP expression varied depending on element, but all constructs exhibited a reproducible expression pattern.

Heat shock of transgenic *Xenopus*

Xla.Tg(Xtr.pax8:GFP;hsp70:klf15-2A-mCherry) transgenic *X. laevis* at tailbud stage 26 were treated at 34ºC for 15 min, followed by 15 min at 14ºC. These steps were repeated three times and embryos were incubated at 18ºC. Normally-developed heat shock-treated embryos were then sorted by mCherry positivity or negativity (SI Appendix, Fig. S6A). Sorted embryos were used for qPCR and ChIP-qPCR analysis. In the case of EnR fused Klf15 transgenesis, all mCherry positive *Xla.Tg(Xtr.pax8:GFP;hsp70:EnR-klf15-DBD2-2A-mCherry)* transgenic *X. laevis* showed severe development defects. Therefore, we treated two cycles of 34ºC for 15 min and 14˚C for 15 min. Following observations of regenerating nephric tubules, all embryos were subjected to reverse transcription polymerase chain reaction (RT-PCR) to confirm expression of mCherry (SI Appendix, Fig. S6B).

Luciferase reporter assay

HEK293T cells were purchased from RIKEN BRC Cell Bank (Japan). Cells seeded in 48-well plates were transfected with 10 ng of open chromatin element carrying luciferase reporter plasmids, 1 ng effector plasmids, and 10 ng of Renilla luciferase plasmids using jetPEI (Polyplus Transfection SA, Cat#24765-1). Total amount of DNA per well was adjusted to 500 ng pBSK plasmids. Transfected cells were incubated for 48 h, then luminescence signals were measured following the manufacturer's protocol (Promega). Two-tailed unpaired Mann–Whitney t-test was performed using Prisms Software (GraphPad version 9.3.1).

Agonist and antagonist treatment for Adrenoceptor alpha 1A (Adra1a)

Prazosin hydrochloride (Sigma-Aldrich, Cat#P7791) was dissolved in ultrapure water for 0.5 mg/mL stock. Stock solution was diluted in 0.3 × Marc's Modified Ringer's and HEPES buffer (MMR) at a final concentration of 0.04 mg/mL, after which injured embryos were incubated at 18ºC. Buffer was exchanged every 24 hours (8). (-) -Epinephrine. (Sigma-Aldrich, Cat#E4250) was dissolved in dimethyl sulfoxide (DMSO) for 50 mg/mL stock. The stock solution was diluted in 0.3 × MMR for a final concentration of 100 μg/mL and injured embryos were incubated at 18ºC (15–17). A-61603 (Cayman, Cat#17358) was dissolved in DMSO for 100 mM stock. The stock solution was diluted in 0.3 × MMR at a final concentration of 100 nM and injured embryos were incubated at 18ºC with the buffer exchanged every 24 hours.

Immunostaining

Tadpoles were fixed in 3.7% formaldehyde/MEM at 4ºC overnight. After fixation, tadpoles were washed with 100% ethanol, then incubated in a 2% BSA/PBS-t blocking solution. Tadpoles were then transferred to a primary antibody solution. A 1:1000-diluted rabbit anti-phospho-histone H3 (Ser10) antibody (Millipore, Cat#06-570, RRID: AB_310177) was used as the primary antibody. A

1:1000-Alexa 488-conjugated goat anti-rabbit IgG (Invitrogen, Cat#A11001, RRID: AB_2534069) and 1:1000-diluted Alexa 568-conjugated goat anti-mouse IgG (Invitrogen, Cat#A11011, RRID: AB_14315) were used as secondary antibodies. Images were acquired using ApoTome.2 (Axio Zoom.V16; Carl Zeiss). Statistical analysis was performed using Prisms Software (GraphPad version 9.3.1).

In situ hybridization for _klf15_ and _adra1a_

For the *in situ* hybridization probes of *X. laevis, klf15*.L, *klf15.S*, *adra1a.L*, *adra1a.S*, and *h1-5* were amplified from a cDNA pool of *X. laevis* tailbud embryos (stages 35/36). DNA fragments were introduced into the Xho I and Xba I sites of the pCS2 plasmid. *X. laevis* were fixed in 3.7% formaldehyde/MEM at 4°C overnight. After fixation, the embryos were washed with 100% ethanol. *In situ* hybridization was performed using the standard procedure. Signals were photographed using an AxioZoom.V16 with AxioCam MRc cameras (Carl Zeiss).

Quantification of nephric tubule size

We used live embryos to measure the length of the intermediate and area of the nephric tubule for both injured and uninjured sides. ZEN 2 (2.0.0) software based on GFP expression (Carl Zeiss, RRID:SCR_018163) was used for this quantification.

Real-time RT-qPCR

Total RNA for qPCR of *klf6* and *klf15* was extracted from uninjured and injured *Xla.Tg(Xtr.pax8:GFP)* nephric tubules using ISOGEN (NIPPON GENE, Cat# 315-02504). Primers for ornithine decarboxylase 1 (*odc1*), *klf6*, and *klf15* were derived from a previous study (11). Total RNA for qPCR of *adra1a*, *sap25*, *h1-5*, *tmtc2*, *fut4*, and *rab6b* was extracted from heat shock-treated mCherry negative and fluorescence positive whole tadpoles. Primers were designed using Primer3Plus (12). Reverse transcription reactions were performed with random oligo primers (Takara Bio In., Cat#2680A). Transcript levels of target genes were quantified by real-time PCR analysis on a CFX Connect system (Bio-Rad Laboratories) using the KAPA SYBR FAST qPCR Master Mix (KAPA, Cat#KR0389). Statistical analysis was performed using Prisms Software (GraphPad version 9.3.1).

ChIP-qPCR

Genomic DNA was extracted from fluorescence positive *Xla.Tg(hsp70:myc-klf15-2A-mCherry)* and wild-type embryos. Briefly, three whole embryos were crosslinked with 1% formaldehyde for 15 min at room temperature and formaldehyde was quenched by adding glycine to a final concentration of 0.125 M. Chromatin was sonicated to an average size of 0.2–5 kb using Bioruptor UCD-250 (Diagenode). A 1:750 Mouse monoclonal c-Myc (9E10) antibody (Santa Cruz Bio technology Inc. Cat#sc-40, RRID: AB__291323) was added to the sonicated chromatin and incubated overnight at 4ºC on a rotating platform. We retained 1% of chromatin for each ChIP reaction as input DNA. Protein A dynabeads were added to ChIP reactions and incubated for an additional 4 h at 4ºC. Magnetic beads were washed, followed by reversal of crosslinks and DNA purification. Transcript level of target genes was quantified by real-time PCR analysis on a CFX Connect system (Bio-Rad Laboratories) using the THUNDERBIRD SYBR qPCR Mix (TOYOBO, Cat#QPS-201). The open-access database JASPAR (ver. 9) was used to search for potential Klf binding sites (13).

Phylogenetic analysis

Amino acid sequences were obtained from NCBI: *Xenopus laevis* sialidase (XP_018109346.2), *Xenopus tropicalis* histone deacetylase complex subunit SAP25-like (XP_031755455.1), *Bufo bufo* histone deacetylase complex subunit SAP25 (XP_040271799.1), *Myotis myotis* histone deacetylase complex subunit SAP25 (XP_036165974.1), *Homo sapiens* Sin3A associated protein 25 (NM_001348680.2), *Mus musculus* histone deacetylase complex subunit SAP25 (NP_001075431.2),
Rousettus aegyptiacus Sin3A associated protein 25 (KAF6442196.1), *Ictidomys tridecemlineatus* Sin3A associated protein 25 (KAG3259900.1), *Pipistrellus kuhlii* Sin3A associated protein 25 (KAF6367016.1), *Chelydra serpentina* Sin3A associated protein 25, partial (KAG6926129.1),

Homo sapiens neuraminidase 1 (NM_000434.4), *Homo sapiens* neuraminidase 2 (NM_005383.2), *Homo sapiens* neuraminidase 3 (NM_006656.6), *Homo sapiens* neuraminidase 4 (NM_001167600.3),
Mus musculus sialidase-1 precursor (NP_035023.3), *Mus musculus* sialidase-2 (NP_001153635.1),
Mus musculus sialidase-3 (XP_030098621.1), *Xenopus tropicalis* sialidase-1 (XP_002941345.1), *Xenopus tropicalis* sialidase-4-like (XP_031751650.1), and *Myotis myotis* neuraminidase 1 (KAF6276702.1). MUSCLE algorithm and UPGMA algorithm mounted on MEGA X software were used to align sequences and reconstruct the phylogenetic tree (14).

A

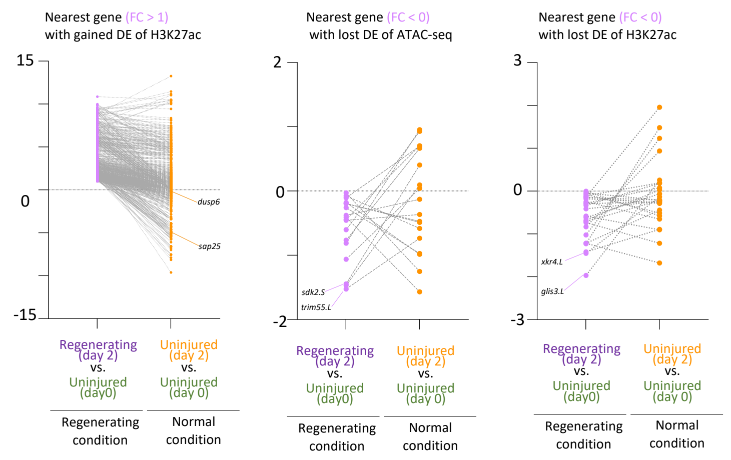

B

Gene	Regenerating (2 day) vs. Uninjured (0 day)	Uninjured (2 day) vs. Uninjured (0 day)	PValue
**gene-LOC108712571**<br>**cat eye syndrome critical region protein 2 (CECR)**	2.92539459888974	-2.96607603963267	6.55354002569062E-16
**gene-LOC108712787**<br>**adrenoceptor alpha 1A (ADRA1A)**	1.9597805653068	-0.253854435963227	0.447149621071245
**gene-LOC108717144**<br>**RAB6B, member RAS oncogene family**	1.78049088568271	-1.03438345778519	0.0652849240114001
**gene-LOC108710013**<br>**fucosyltransferase-4 (FUT4)**	1.73632575796544	-0.637251425076122	0.368616768176773
**gene-LOC108711733**<br>**A.superbus venom factor 1**	1.6806124282414	-1.20012575577342	2.44393693625387E-06
**gene-LOC108711801**<br>**Sin3A associated protein 25 (SAP25)**	1.25870503612927	-5.07252966914263	0.163315018078803
**gene-LOC495060**<br>**dual specificity phosphatase 6 S homeolog (DUSP6)**	1.21124795389492	-0.142402487828901	0.00273727234328497
**gene-LOC108716608**<br>**uncharacterized**	1.1962114699567	-5.0904227774913	0.140967951756267
**gene-LOC108703808**<br>**histone H1B L homeolog (H1-5)**	1.09565787818485	-3.19447270797358	0.665416854936768
**gene-tmtc2.S**<br>**transmembrane O-mannosyltransferase targeting cadherins 2 (TMTC2)**	1.05390825194529	-0.724014596347332	0.0584848901160807

Fig. S1. (A) Upregulated genes nearest to gained H3K27ac DEs of ChIP-seq and downregulated genes nearest to lost DEs of ATAC-seq and H3K27ac ChIP-seq. Genes nearest to gained DEs and lost DEs were extracted using ChIPpeakAnno. Upregulated and downregulated genes were then extracted (purple circles). Their counterpart genes in normal conditions (uninjured tubules [day 2] vs. uninjured tubules [day 0]) are plotted as orange circles. *sap25* and *dusp6* were the nearest genes of gained H3K27ac DEs as observed in gained ATAC-seq DEs, while others, such as *adra1a*, are not listed. Since H3K27ac enrichment on their elements was observed on day 2, it may become further enriched around days 3–4. Genes downregulated in the regenerating tubule and upregulated under normal conditions may be unnecessary. (B) List of genes closest to gained DE genes upregulated in regenerating nephric tubules and suppressed under normal conditions.

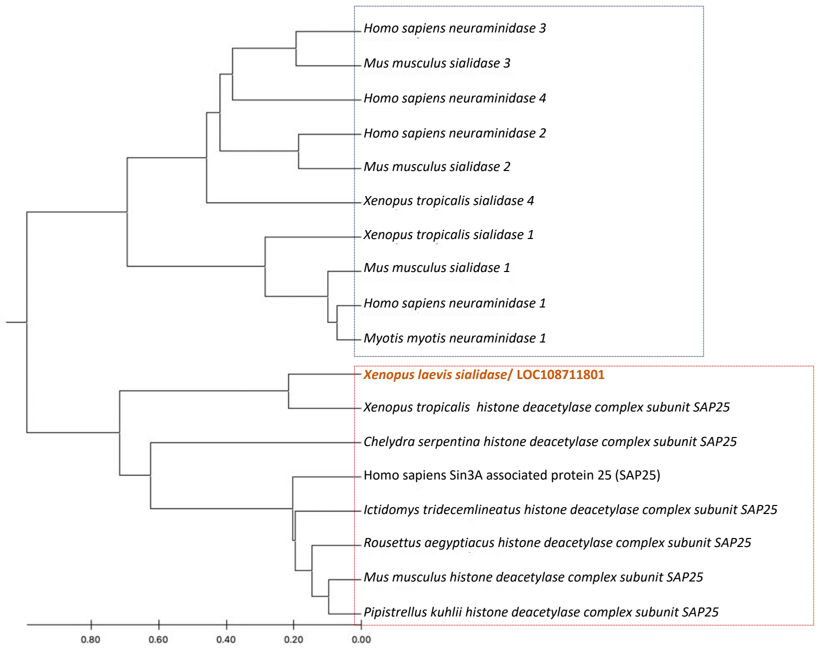

Fig. S2. Gene-LOC108711801 belongs to the SAP25 family. Gene prediction suggests that Gene-LOC108711801 is a sialidase isoform X1 or signaling mucin HKR1-like, while phylogenetic analysis indicates that gene-LOC108711801 is closer to SAP25.

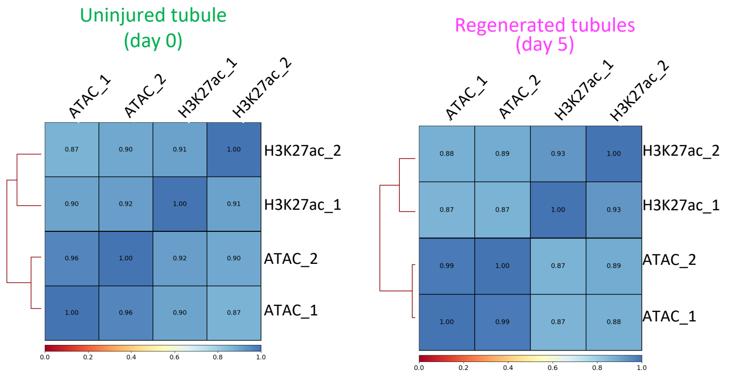

Fig. S3. Correlation analysis of ATAC-seq and ChIP-seq peaks. Hierarchically clustered correlation matrix of ATAC-seq and ChIP-seq peaks and their replicates (rep1 and rep2). Pearson correlations were calculated in deepTools (3.5.0) module multiBamSummary with default parameters. (B) Number of gained peaks and lost peaks in regenerating tubules (day 2).

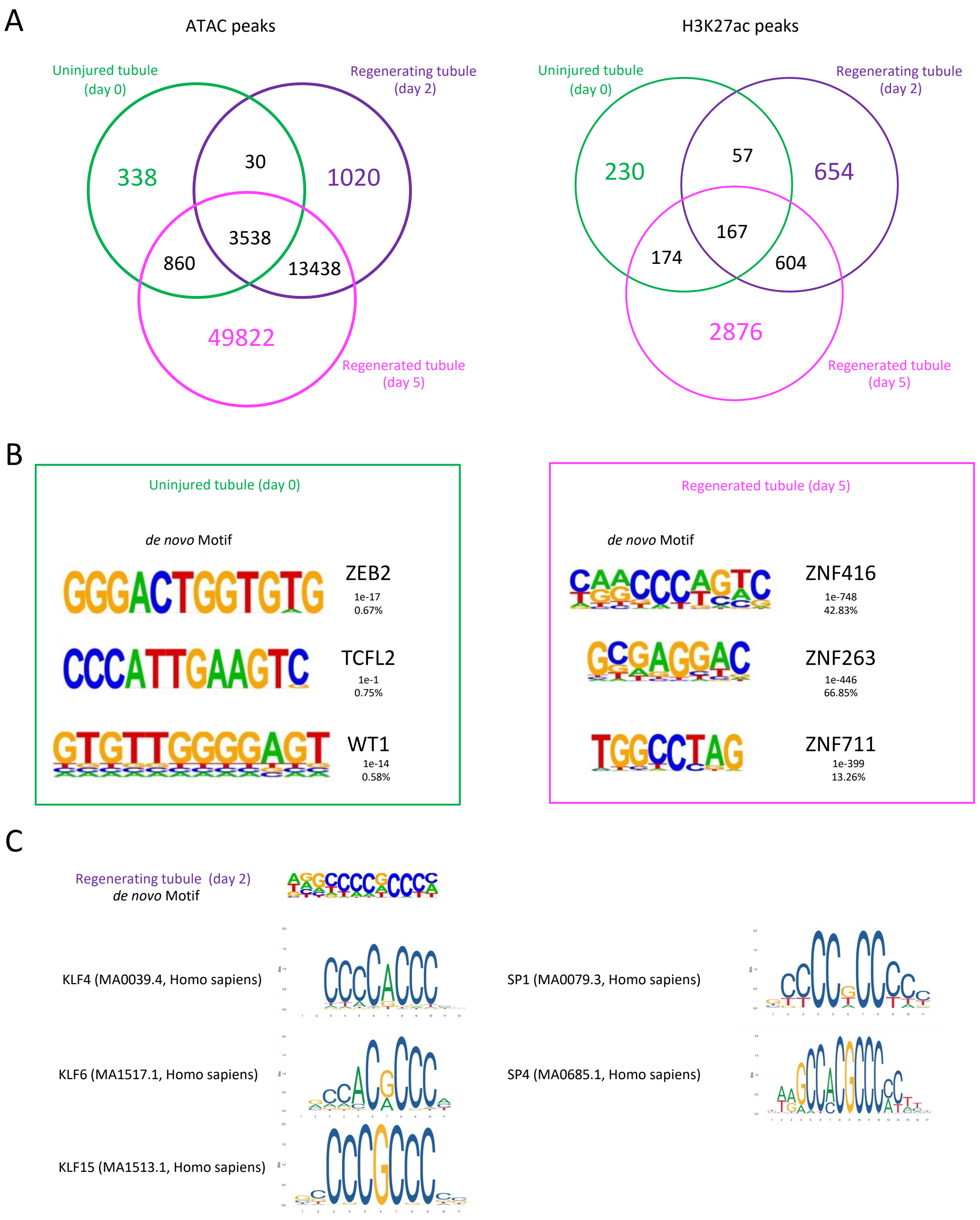

Fig. S4. (A) Number of shared and unique peaks in ATAC-seq and H3K27ac ChIP-seq. (B) *De novo* identification of uninjured tubules (day 0) and regenerated tubules (day 5) specific to open chromatin elements and best matches to known motifs. P values for motif enrichment and percentage of regions containing motifs are indicated to the right of sequence logos. (C) Comparison of regenerating tubule specific to *de novo* motif and known Klf motifs derived from the JASPR database.

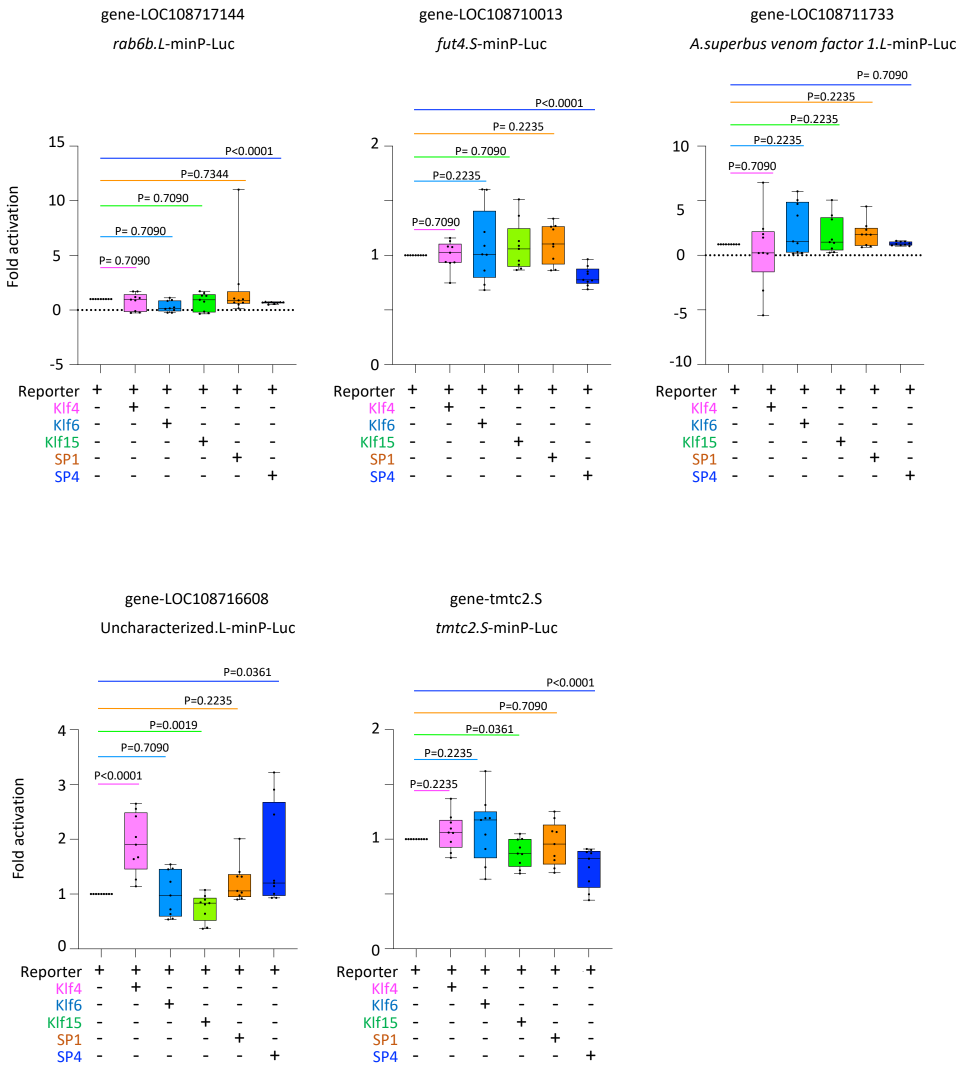

Fig. S5. Luciferase reporter assay for *rab6b.L*, *fut4.S*, *A. superbus venom factor 1.L*, uncharacterized protein.L, and *tmtc2.S* associated gained DE open chromatin elements. Klf4, Klf6, Klf15, SP1, and SP4 were cotransfected with reporter plasmids into HEK293T cells. Two-tailed unpaired Mann–Whitney t-test where whiskers show minimum and maximum and contain all points.

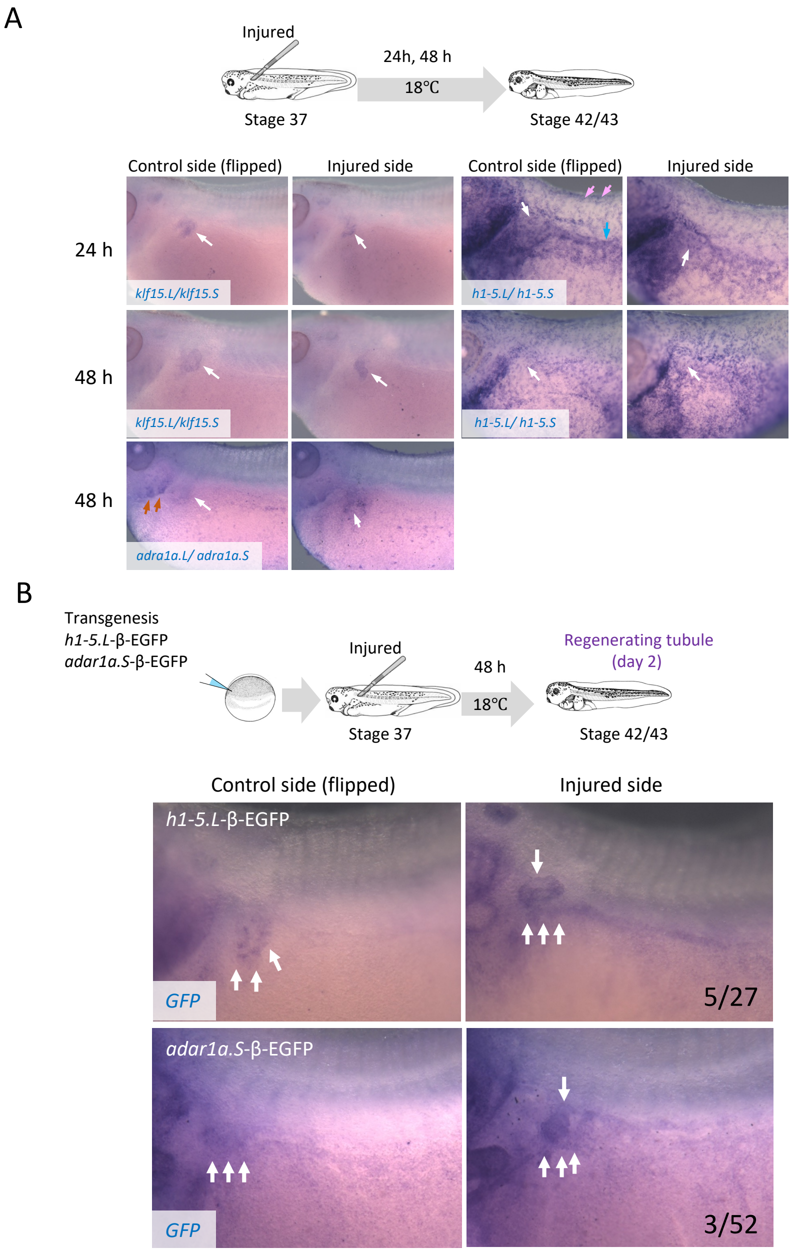

Fig. S6. (A) Expression of *klf15*, *adra1*, and *h1-5* in *X. laevis*. The regenerating tubule expressed *klf15*. Although *adra1a* showed a slightly stronger expression in gills, its expression is broad. The expression of *h1-5* was detected in the nephron, the intersomitic vessels, and the posterior cardinal vein. The white arrows indicate the nephric tubule. The orange arrows indicate the gill. The pink arrows indicate the intersomitic vessels. The blue arrow indicates the posterior cardinal vein. (B) Open chromatin element in regenerating nephric tubule response to injury signal. GFP reporter constructs carrying open chromatin elements associated with *adra1a.S*-β-EGFP and *h1-5.L*-β-EGFP were subjected to transgenesis. All reporter-injected embryos underwent injury on the left side at stage 37. Arrow indicates regenerating nephric tubule and GFP signals. N indicates the number of scored tadpoles. *adra1a.S*-β-EGFP: five tadpoles showed a stronger GFP signal on the injured side compared with the uninjured control side. *h1-5.L*-β-EGFP: five tadpoles showed a stronger GFP signal on the injured side compared with the uninjured control side.

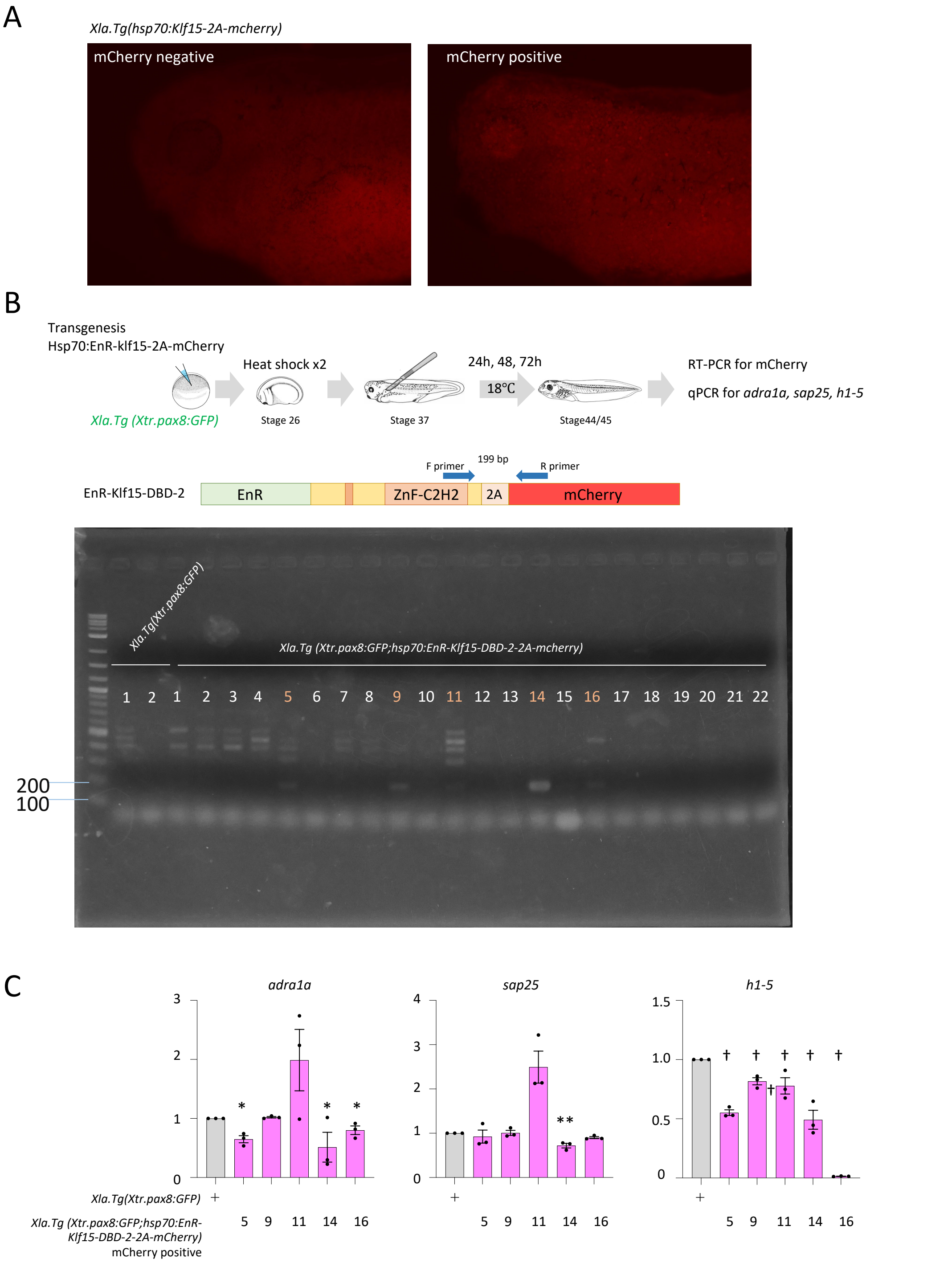

Fig. S7. (A) Fluorescence mCherry signal in *Xla.Tg(hsp70:klf15-2A-mCherry)*. (B) RT-PCR analysis for identifying mCherry mRNA expressing *Xla.Tg (Xtr.pax8:GFP;hsp70:klf15-2A-mCherry)*. (C) Five out of five *mCherry* mRNA positive tadpoles showed the decreased expression of *h1-5*, and three out of five showed the decreased expression of *adra1a*, while only one out of five tadpoles showed the decreased expression of *sap25* at 72 h after injury (†, * and **, respectively).

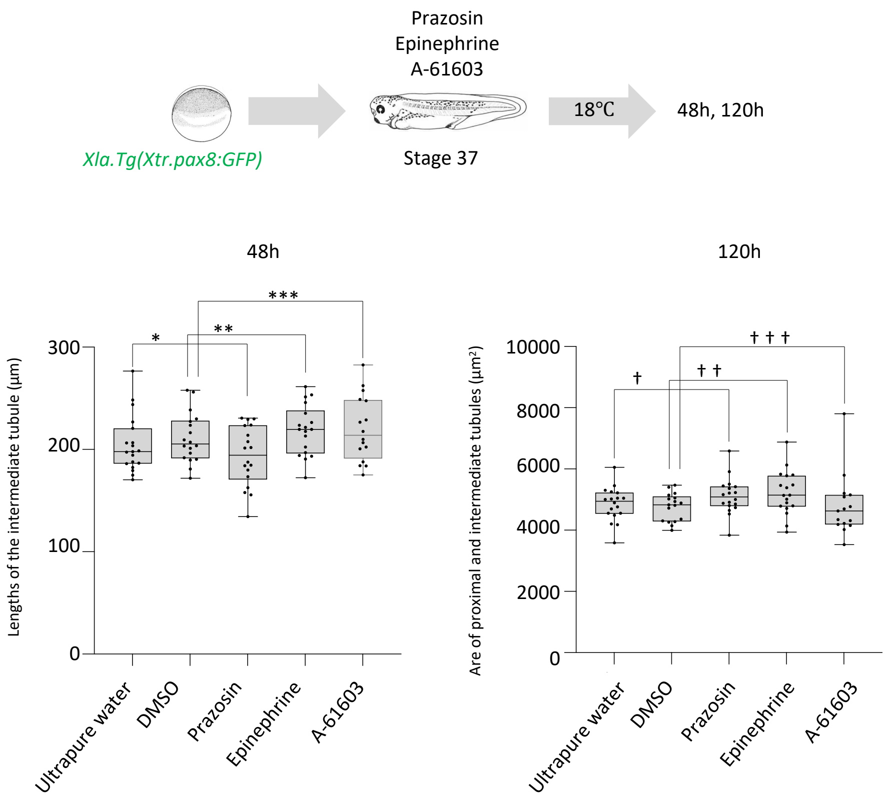

Fig. S8. Uninjured *X. laevis* embryos were treated with epinephrine, A-61603, and prazosin. The length of the left side intermediate tubule and the area of the nephric tubule were measured at 48 h and 120 h. No significant difference between control and epinephrine/A-61603/prazosin-treated tadpoles was observed. Two-tailed unpaired Mann–Whitney t-test: *p = 0.4612; **p = 0.3231; ***p = 0.5060, †p = 0.2262; ††p = 0.0717; †††p = 0.5508.

Dataset S1 (separate file). RNA-seq data of regenerating tubule [day 2] vs. uninjured tubule [day 0].

Dataset S2 (separate file). Genes associated with the gained DEs.

Dataset S2 (separate file). Primer sequence lists.

SI References

1. M. R. Corces, *et al.*, An improved ATAC-seq protocol reduces background and enables interrogation of frozen tissues. *Nat. Methods* **14**, 959–962 (2017).
2. J. Brind'Amour, *et al.*, An ultra-low-input native ChIP-seq protocol for genome-wide profiling of rare cell populations. *Nat. Commun.* **6**, 1–8 (2015).
3. F. Ramírez, *et al.*, deepTools2: a next generation web server for deep-sequencing data analysis. *Nucleic Acids Res.* **44**, W160–W165 (2016).
4. H. Ogino, M. Fisher, R. M. Grainger, Convergence of a head-field selector Otx2 and Notch signaling: a mechanism for lens specification. *Development* **135**, 249–258 (2008).
5. N. Suzuki, K. Hirano, H. Ogino, H. Ochi, Arid3a regulates nephric tubule regeneration via evolutionarily conserved regeneration signal-response enhancers. *Elife* **8**, 1–28 (2019).
6. G. N. Wheeler, F. S. Hamilton, S. Hoppler, Inducible gene expression in transgenic Xenopus embryos. *Curr. Biol.* **10**, 849–852 (2000).
7. K. L. Kroll, E. Amaya, Transgenic Xenopus embryos from sperm nuclear transplantations reveal FGF signaling requirements during gastrulation. *Development* **122**, 3173–3183 (1996).
8. M. P. O'Daniel, M. L. Petrunich-Rutherford, Effects of chronic prazosin, an alpha-1 adrenergic antagonist, on anxiety-like behavior and cortisol levels in a chronic unpredictable stress model in zebrafish (Danio rerio). *PeerJ* **2020**, 1–24 (2020).
9. S. Mori, Y. Moriyama, K. Yoshikawa, T. Furukawa, H. Kuroda, β-adrenergic signaling promotes posteriorization in Xenopus early development. *Dev. Growth Differ.* **55**, 350–358 (2013).
10. W. K. Martin, *et al.*, High- throughput video processing of heart rate responses in multiple wild-type Embryonic Zebrafish per imaging field. *Sci. Rep.* **9**, 1–14 (2019).
11. Y. Gao, *et al.*, Krüppel-like factor family genes are expressed during Xenopus embryogenesis and involved in germ layer formation and body axis patterning. *Dev. Dyn.* **244**, 1328–1346 (2015).
12. A. Untergasser, *et al.*, Primer3-new capabilities and interfaces. *Nucleic Acids Res.* **40**, 1–12 (2012).
13. J. A. Castro-Mondragon, *et al.*, JASPAR 2022: the 9th release of the open-access database of transcription factor binding profiles. *Nucleic Acids Res.* **50**, D165–D173 (2022).
14. S. Kumar, G. Stecher, M. Li, C. Knyaz, K. Tamura, MEGA X: Molecular evolutionary genetics analysis across computing platforms. *Mol. Biol. Evol.* **35**, 1547–1549 (2018).
